# Measurement of Prosocial Reasoning among Chinese Adolescents

**DOI:** 10.1100/2012/174845

**Published:** 2012-07-31

**Authors:** Frank H. Y. Lai, Andrew M. H. Siu, Chewtyn C. H. Chan, Daniel T. L. Shek

**Affiliations:** ^1^Department of Rehabilitation Sciences, The Hong Kong Polytechnic University, Hong Kong; ^2^Department of Applied Social Sciences, The Hong Kong Polytechnic University, Hong Kong

## Abstract

This study attempted to develop a standardized instrument for assessment of prosocial reasoning in Chinese populations. The Prosocial Reasoning Objective Measure (PROM) was translated, and a two-stage study was conducted to evaluate the psychometric properties of the translated instrument. The content validity, cultural relevance, and reading level of the translated instrument were evaluated by an expert panel. Upon revisions according to the expert opinions, the Chinese PROM demonstrated good content validity, “good-to-very good test-retest” reliability, and internal consistency. However, only partial support to the convergent validity of the Chinese PROM was found. In the first stage of the study (*n* = 50), the PROM scores had high positive correlations with empathy and negative correlations with personal distress and fantasy. These results were consistent with theoretical expectations, although this is also a concern that empathy had a close-to-unity correlation with PROM score in the small sample study of stage 1. In the second stage of the study (*n* = 566), the relationship between PROM scores and prosocial behavior appeared to be weak. Results suggest that there were many personal, family, or social factors that were linked to prosocial behavior, and prosocial reasoning might only contribute to a small proportion of variation in prosocial behavior among adolescents.

## 1. Introduction

Moral reasoning is defined as the process of judging right and wrong and is regarded as the force behind moral action [1]. As child progress to adolescence, their moral reasoning changes from the “self-focused” or “self-centered” status/mentality (what feels good to me is right) to a stance in which social approval guides both reasoning about justice and about doing good. Moral reasoning becomes more sophisticated as a child reaches adolescence, an empathic orientation stage in which they often express sympathetic concerns for others. The empathic orientation could further develop into the internalized value orientation stage in late adolescence or early adulthood, which is defined as an orientation to an internalized responsibility, duty, or need to uphold the laws and accepted norms or values [2, 3]. The young person eventually develops an individualized ethical code for directing their moral behavior.

Eisenberg adopted the stages of moral development of Kohlberg and examined how prosocial (moral) reasoning is linked to prosocial behavior like sharing, cooperating, helping, volunteering, and comforting others [4]. Based on the theories of moral development [1] and empirical studies, Eisenberg further refined the five stages of prosocial reasoning [5]. (1) Hedonistic (self-focused) orientation: the respondent only cares for oneself, and any apparent altruistic behavior is motivated by selfishness, for example, “I'll help them because they'll help me in future” (reciprocity), or simply because the children likes the person they are helping. (2) “Needs of others” orientation: it addresses the needs of others who are being recognized only to a limited extent. The needs of the specific situation are being addressed without a genuine sense of empathy (3) Stereotyped and approval-focused orientation: adolescents act in a way that will make them popular or liked by others, for example, lending a helping hand in order to impress others. When they are asked to explain their behavior, they tend to use stereotyped portrayals of good and bad behavior. (4a) Empathic orientation: adolescents start to show genuine empathy by putting themselves in the shoes of others and begin to report feelings of genuine guilt when considering their own actions. (4b) Transitional level: adolescents explain their actions by referring to wider social values and the need to protect the dignity and self-esteem of others. (5) Internalized orientation: the adolescents have a comprehensive set of values and understand their responsibilities towards others. They harbor self-respect that they can only maintain by behaving with a duty of care towards others. The person's desire to live up to their own set of principles is also a motivating factor for the development of prosocial behavior.

The assessment of moral reasoning was often conducted by using moral dilemmas—hypothetical situations in which people are required to make difficult decisions. During the assessment, it is more significant to examine the reasoning behind rather than the actual choice made. In line with the approach used by Kohlberg [1], Eisenberg and associates [5, 6] presented ethical dilemmas for assessing the stage of development of the prosocial moral reasoning in children and adolescents. They asked respondents to take up the role of someone else and decide whether to act out of self-interest or in the interests of others.

To date, many of the previous studies on prosocial moral reasoning were conducted by using interview measures of moral reasoning. In recent years, some self-completed measures of moral reasoning have been designed to assesses prosocial reasoning, which could be more efficiently and effectively administrated to larger research samples [7–9]. Based on Eisenberg's prosocial moral reasoning interview measure [2], Carlo and associates developed a paper-and-pencil measure named Prosocial Reasoning Objective Measure (PROM) [10]. The PROM is a self-completed questionnaire which assess prosocial reasoning using moral dilemmas, in which a person's needs/desires conflict with those of needy others, with formal obligations minimal or absent [11]. It is one of the few standardized instruments designed for measuring prosocial reasoning, which takes approximately 20 to 30 minutes to complete.

The original English PROM has been administered to child, adolescent, and adult samples, and its psychometric properties were promising [12]. The test-retest reliability of the standard 5-story version was good, with coefficients ranging from  .70 to  .79 [10]. Internal consistency was fair to acceptable, with Cronbach's *α* ranging from  .56 to  .78. For the 7-story version, internal consistency (*α*) ranged from  .61 to  .85 for the five types of reasoning, but no test-retest reliability was reported by Carlo et al. [11].

Studies of construct validity of the 5-stories version showed that sympathy was positively associated with higher levels of prosocial moral reasoning in PROM and tended to be negatively related to lower levels of moral reasoning (i.e., hedonistic and approval-oriented reasoning). These findings are consistent with prior empirical findings [11, 13, 14] that self-reflective types of moral reasoning often reflect other-oriented cognitions and feelings. These psychometric studies provided evidence that the 5-story version PROM is a reliable and valid measure of prosocial moral reasoning for adolescents.

The PROM has been translated into Portuguese, Spanish, Korean, and Tagalog (Philipino), and there were slight modifications of the test stories for different language versions. A Chinese version of 5-story PROM had also been developed in Taiwan, but no validation study of the Chinese version has been completed so far. Culture plays an important part in how people understand and define prosocial behavior in a society. It is therefore essential to examine the cultural relevance and validity of the PROM if it is used within a Chinese population.

This study attempted to evaluate the psychometric properties of the Chinese Prosocial Reasoning Measure (Chinese PROM), that is, to assess whether the Chinese version PROM is culturally relevant, reliable, internally consistent, and valid. The convergent validity of PROM will be investigated by studying its relationships with measures of empathy and prosocial as well as antisocial behavior.

There are several reasons in developing a standardized instrument for measuring prosocial reasoning. First, the stage theory of prosocial moral reasoning has long been used in describing moral development. It is of empirical interest to examine if measures of prosocial reasoning (like the PROM) could be developed by using the stage theories. Second, there is a growing research interest in studying the factors underlying the development of prosocial behavior [15, 16], but the lack of a standardized instrument for efficient assessment of prosocial reasoning could be a major barrier to further research in this area [17]. Third, the testing of moral reasoning has long been assessed through ethical or moral dilemmas by using the semistructured interview format. The PROM replaces the interview method with self-completed questionnaire format, and there is a need to examine if this format of assessment could be as valid and reliable. Fourth, there is a need to examine the cultural relevance and psychometric properties when the PROM is used with Chinese adolescents. Culture clearly makes a substantial contribution to the development of prosocial development in Chinese populations [18, 19].

## 2. Method

This study attempted to conduct a validation study of a Chinese version of Prosocial Reasoning Objective Measure (Chinese PROM). The study comprises two stages. In the first stage, we evaluated the content validity and cultural relevance through expert panel review and estimated test-retest reliability and internal consistency of the Chinese version of PROM by using a small sample (*n* = 50). We also examine the relationship between PROM scores and empathy. In the second stage, we collected a large sample (*n* = 566) to examine the validity of the PROM by correlating its scores with prosocial and antisocial behavior.

## 3. Study 1

### 3.1. Expert Panel Review

The original English version of the PROM was translated into Chinese by a professional translator. A group of 5 experts in youth development research was invited to review the translated Chinese PROM. On a self-completed questionnaire, the experts were requested to use a five-point scale (from “strongly disagree” to “strongly agree”) to rate how far the test scenarios and items were relevant to test prosocial reasoning, and how these five scenarios are representative of situations for assessing prosocial reasoning in young people. A second expert panel, which was composed of three secondary school teachers and two social workers, assesses the clarity of presentation of the test scenarios and evaluates if the story contents could be easily understood by young people at a reading level of grade 6 or above.

For both expert panel reviews, a mean score of 4.0 (agree) was selected as the criterion for the cut-off score of a clear presentation, good-content understandability, and the selected scenario is relevant to young people. Justifications would be requested for items that are considered irrelevant, and recommendations on modifications of items that needed revision would also be requested. Refinement and finalization of items were made according to opinions of these panels' reviews.

### 3.2. Study of Reliability and Convergent Validity

#### 3.2.1. Participants

A convenient sample of young people from early to mid-adolescence was recruited through parents' network and a youth service agency, if they are Chinese, aged between 12 and 16 years old, are full time secondary school students, can read and write Chinese. A sample of 50 participants (25 males and 25 females) was recruited. Their mean age is 13.5 (SD = 1.43), and they were secondary 1 to 5 students. Twenty-four of them were in junior high school, while 26 subjects were in high school. They came from 10 different schools.

#### 3.2.2. Instruments

**Prosocial Reasoning**

The Prosocial Reasoning Objective Measure (PROM) was used to examine prosocial moral reasoning in young people and adults [10]. The PROM requested respondents to read stories about people who need help from others and then decide whether they would offer help and the reasoning behind it. The dilemmas in the stories are designed to invoke a conflict between the actor's needs, wants, and desires and those of another (or others). The Chinese short version uses 5 stories that are translated from the short version of the English PROM: (1) donating blood to needy others versus losing time and money at work and school, (2) choosing to get an injured child's parents versus going to a friend's party, (3) continuing to stay and play in one's own backyard versus going to try and stop a bully that is picking on a peer, (4) helping disabled children strengthen their legs by teaching them to swim versus practicing for a swimming contest to win the prize in cash, (5) keeping food after a flood versus giving some food to others who had none.

After reading each story, respondents were asked to rate on a scale of one to five (from “greatly important” to “not important”) on how important each of the five reasons was in deciding what the character should do. The five reasons reflected the five types of prosocial reasoning of hedonistic, needs-oriented, stereotyped, approval-orientated, and internalized. The overall weighted PROM score is an overall score that reflects the development or maturity of prosocial moral reasoning. According to the PROM manual, the overall weighted PROM score is the sum of proportion scores of internalized reasoning multiplied by 3, needs-oriented and stereotypic reasoning multiplied by 2, and hedonistic and approval-oriented reasoning.

PROM stories were slightly modified for use with different age groups and cultural groups [5, 12]. The psychometric properties of PROM have been reported in studies with students from middle childhood to early adulthood. The results have been well elaborated [20]. The test-retest reliability of PROM ranged from  .70 to  .79, while Cronbach's *α* ranged from  .56 to  .78 [10].

**Empathy and Related Constructs**

The 21-item Chinese Interpersonal Reactivity Index (C-IRI) is a self-reported questionnaire consisting of 3 subscales measuring empathy and related constructs: (1) the Fantasy Scale (FS) that measures the tendency to imaginatively transpose oneself into fictional situations, (2) the Empathy Scale (ES) that measure the tendency to experience feelings for sympathy and perspective/role taking, (3) Personal Distress Scale (PDS) that measures the tendency to experience distress and discomfort in response to extreme distress in others. Participants are requested to indicate the degree they agree with each item by using a 5-point Likert-type scale, which varied from 0 (*does not describe me well*) to 4 (*describes me very well*). A higher score in the three subscales represents a higher tendency in each aspect of empathy. The C-IRI possessed acceptable psychometric properties in Chinese adolescent samples [21], and empathy-related constructs can be conceptualized as a convergent construct with prosocial moral reasoning.

#### 3.2.3. Procedures

A cover letter, a research information sheet, and a consent form were sent to the potential participants. The documents described the background and purpose of the study. The participants were instructed to sign on a consent form if they agreed to participate in this study and returned the completed questionnaires to their teachers or parents.

To assess test-retest reliability, 25 of the 50 participants completed the Chinese PROM twice, with an interval of one week in between. The internal consistency estimates (Cronbach's *α*) were obtained from the data set of all participants (*N* = 50). For the data collection on convergent validity, a group of 50 full time secondary school students was recruited using the same selection criterion. The participants were requested to complete the Chinese PROM, the C-IRI, and correlations between the two measures were estimated.

## 4. Study 2

This stage of the study aims to recruit a larger sample for studying convergent validity between PROM scores and adolescent prosocial and antisocial behavior, and also sex differences in PROM scores.

### 4.1. Instruments

The Prosocial Reasoning Objective Measure (PROM), that was used in stage 1, was also used in this stage of the study.

**Prosocial Behavior**

The Adolescent Behavior Questionnaire (ABQ) is generic Chinese instrument designed for measuring the prosocial and antisocial behavior of adolescents [22]. The prosocial scale assesses normative acts and altruistic acts, while the antisocial scale covers rule-breaking, challenging, or aggressive behavior in school, home, and social settings. The respondents were asked to report the frequency of 65 behaviors performed in the past year on a 7-point Likert-type scale (1 = none, 2 = 1-2 times, 3 = 3-4 times, 4 = 5-6 times, 5 = 7-8 times, 6 = 9-10 times, and 7 = more than 10 times). The ABQ possesses two general scales of antisocial/delinquent Behavior (DB) scale and prosocial behavior (PB) scale. The Adolescent Behavior Questionnaire (ABQ) score is the difference between the mean scores of prosocial behavior and antisocial behavior scales. It indicates how far a young person's behavior is antisocial (negative scores) or prosocial (positive scores).

### 4.2. Participants

A convenient sample of 566 young people in mid to late adolescence was recruited from 36 secondary schools. The ages of the participants ranged from 14 to 22 years old (*M* = 16.2, SD = 1.1), but 79% were between 15 and 17 years old. There were more females (67.7%) than males 32.3% in the sample. Most of the participants were studying secondary 4 (52.2%) or secondary 5 (42.3%), and a few were studying secondary 6 (5.4%). Teachers help to carry out the study. It may involve a certain extent of social desirability and the ethical consideration.

### 4.3. Procedures

All potential participants were given a briefing about the purpose and information about the study (based on a research information sheet) by class teachers. All participants (students) who were willing to participate in the study would be requested to sign on a consent form for volunteer participation in the study. For potential participants who are younger than 18 years, an invitation letter would be sent to their parents. Parents who endorsed their children to join the study are requested to sign on and return a consent form to the school. Ethical approval of this study had been obtained from the Departmental Research Committee of our university. The school sent all the consent forms and completed questionnaires back to the researchers for record and analysis.

## 5. Results

### 5.1. Expert Review of Content Validity

A total of 11 content experts were invited to comment on the content validity, cultural relevance, and reading level of the translated instrument (Chinese PROM). The experts are professionals in education, social work, clinical psychology, and education. Six of the experts are academics and experienced researchers on the youth development in Hong Kong. Five of the experts were frontline workers in providing educational, social work, or counseling service to young people.

On the whole, the experts agreed that the contents of five test scenarios were relevant to assessment of prosocial reasoning (mean rating of 4.46 out of 5) and representativeness of stories for testing prosocial reasoning (mean rating of 4.49 out of 5). However, the experts found that the reading level and cultural relevance of scenario 2 were not satisfactory (mean rating were 3.73 and 3.76, resp.). The experts suggested that most adolescents in Hong Kong have a mobile phone, and the young person in scenario 2 could seek help by using his/her mobile. Thus, the story was changed from “going to her home and told her parent to come for help” to “stay with her till her parent comes.” The experts also gave a number of suggestions to further improve the presentation of the test scenarios, including: (1) use of terms that can be more easily understood by young people, (2) improve the quality of translation, (3) simplification of sentences, (4) clarification of meaning, and (5) amend grammatical errors.

### 5.2. Reliability

The internal consistency of the PROM subscales and the weighted total ranged from  .74 to  .93 (Cronbach's *α*), while the test-retest reliability ranged from  .75 to  .88 (ICCs) (Table 1). The reliability estimates are regarded as ranging from “acceptable” to “satisfactory.” The reliability estimates were much higher than that reported in the study by Carlo in 1992 [10].

### 5.3. Relationship between PROM Scores and C-IRI

Based on the small sample collected in stage 1 (*n* = 50), the results showed that the PROM scales and subscales had significant correlations with the C-IRI subscales (Table 2). The hedonistic and approval-oriented subscales in PROM had a different pattern of correlations from the other three subscales. Both the hedonistic and approval-oriented subscales had significantly positive correlations with fantasy (*r*_hedonistic_ = .55, *r*_approval-oriented  _ = .61) and personal distress subscales (*r*_hedonistic_ = .61, *r*_approval-oriented_ = .68). The pattern of correlation of these two subscales was opposite to that between the needs-oriented, stereotypic, and internalized subscales with the C-IRI subscales. These three subscales had significant negative correlations with fantasy and personal subscales of C-IRI and significant positive correlation with empathy subscale of the C-IRI. The overall weighted PROM score had significant negative correlation with fantasy (*r* = −.77) and personal distress (*r* = −.80) subscales of C-IRI and significant positive correlation with empathy subscales (*r* = .92).

### 5.4. Relationship between PROM Scores and Prosocial/Antisocial Behavior

On the whole, PROM scores were not strongly related to antisocial, prosocial, or adolescent behavior (Table 3). Among the five subscales, only hedonistic and internalized reasoning subscales showed low significant correlations with adolescent behavior. Hedonistic reasoning subscale had significant positive correlation with antisocial behavior (*r* = .14, *P* < .01), and negative correlations with prosocial behavior (*r* = −.10, *P* < .05) and adolescent behavior (*r* = −.17, *P* < .01). Internalized reasoning subscale had low significant correlation with adolescent behavior (*r* = .10, *P* < .05). Overall weighted PROM score had significant correlations with prosocial behavior (*r* = .10, *P* < .05) and adolescent behavior (*r* = .12, *P* < .05).

### 5.5. Gender Differences

Multivariate ANCOVA was conducted to examine gender differences in PROM weighted total and subscales, with age as covariate (Table 4). Multivariate test results showed that age was not a significant covariate, and there were no differences in the profile of five PROM subscales between males and females. There were, however, significant differences in hedonistic and stereotypic reasoning subscales between males and females. Males had significant higher hedonistic reasoning (*F* = 13.06, *P* < .001) than females, and females had significantly higher needs-oriented reasoning (*F* = 7.54, *P* < .01) than males.

## 6. Discussion

A Chinese version of the PROM was developed in this study for measuring prosocial moral reasoning in young people. Through an expert panel review, the translated instrument had good-content relevance and representativeness. The expert panel also identified some issues in the translation, presentation, and reading level of the test scenarios, and modifications were done according to the expert opinions. The Chinese PROM weighted overall and subscales had “acceptable” to “very good” test-retest reliability and internal consistency, which is significantly higher than studies of the original English version. The results supported that the Chinese PROM has good-content validity and reliability.

The study of convergent validity yields mixed results. High levels of prosocial reasoning (PROM weighted overall) were associated with high levels of empathy but low levels of fantasy and personal distress. In particular, the correlation between empathy and PROM weighted overall score was close to unity. This implies that young persons with high empathy are very likely to reach an internalized stage of prosocial reasoning. It is interesting to find that hedonistic and approval-oriented reasoning is strongly and negatively associated with empathy, while all other reasoning types had positive correlation with empathy. This implies young persons with high hedonistic or approval-oriented reasoning are less likely to apply empathy. Since the developmental theories postulated that empathy precedes the development of higher level of prosocial reasoning [23–25], the results support that hedonistic and approval-oriented are the less mature types of prosocial reasoning. Self-reflective types of moral reasoning are often elicited by the tendency to feel concerned for others. The positive relationship between need oriented and empathy is not consistent with theoretical expectations or empirical findings in a Western culture. Need oriented is also regarded as a less mature type or reasoning in the stage theories of prosocial development, but it could be regarded as a socially desirable response to the needs of others in Chinese culture. On the other hand, undertaking prosocial behavior due to the needs of the specific situation could be regarded as socially desirable in the Chinese culture. In fact, a needs-oriented type of reasoning could exist with or without a genuine sense of empathy.

Personal distress is considered as primitive and self-focused empathic reaction [25]. Our findings echoed previous findings that personal distress was negatively related to both stereotypic and internalized reasoning whilst positively related to approval-oriented reasoning [10, 23]. Fantasy has a pattern of correlation with prosocial reasoning that is similar to that of personal distress. Fantasy could be a precursor to empathy, but it could be a barrier to apply empathy or moral reasoning if fantasy stays very strong in social interaction. It is therefore reasonable that fantasy has a negative correlation with overall score of prosocial reasoning and positive correlations with hedonistic and approval-oriented reasoning. In general, the results indicate that a high level of personal distress and fantasy is associated with lower levels of maturity in prosocial reasoning. It appears that personal distress and fantasy, as emotional and imaginative aspects of reacting to others, could be barriers to the application of prosocial reasoning.

The low correlations between PROM scores and measures of antisocial or prosocial behavior did not provide strong support to the convergent validity of PROM. The PROM overall weighted score had low but significant correlations with prosocial and overall adolescent behavior (a score of mean prosocial behavior minus antisocial behavior). Among the subscales, only hedonistic reasoning had significant and low positive correlation with antisocial behavior and low negative correlations with prosocial and overall adolescent behavior. Internalized reasoning had low and significant correlation with overall adolescent behavior. The first implication of the results is that prosocial reasoning may have little influence on prosocial behavior. Adolescent prosocial behavior could be shaped by a wide range of family and social factors, as well as their own development in interpersonal competence [26]. Further research on predicting prosocial behavior may need to include social influence factors other than prosocial reasoning. Second, the studies also showed that proportions of using the five types of reasoning were quite similar (varying from  .17 to  .22) in this sample of high school students. There is a slightly higher proportion of scores (.21 to  .22) for the needs oriented, stereotypic, and internalized, but only slightly higher than hedonistic and approval-oriented reasoning (.17 to  .18). Contrary to expectations of stage theory, the more mature types of prosocial reasoning were not widely adopted by the participants in this late adolescence sample [23, 25]. Further longitudinal studies of prosocial development will be needed to examine the validity of the stage theory, that is, if internalized reasoning increases as young people grow older.

This study is a useful addition to current literature on prosocial reasoning and development. First, it is one of the few studies that investigate the measurement of prosocial reasoning. The PROM is found to be reliable measure of prosocial reasoning. Second, the study also explored how prosocial reasoning is related to convergent constructs of empathy-related constructs and prosocial behavior. The results only provide partial support to the validity of PROM. The results are not consistent with studies conducted in Western cultures and may point to the differences in conceptualization of what is prosocial among cultures. Prosocial reasoning may also be not as important predictor of prosocial behavior in collectivist cultures. The results indicate a need for further replication of the present study. The development of the Chinese PROM enables professionals and researchers to assess prosocial reasoning among young people in an objective and efficient manner. The scale can also be used to evaluate the outcomes of youth development programs.

There are several limitations in this study. First, because of practical limitations, the test-retest interval in the reliability test was one week only. Further study should try to use longer periods of test-retest reliability, such as two to four weeks. Second, the sample in first stage of study was small (*n* = 50). For the second stage of study, most of the participants were from secondary 4 and 5 and a narrow age band. This is a possible reason why age effects were insignificant, which is not in line of results of longitudinal studies of prosocial reasoning conducted in the US. Third, items for monitoring social desirability responses were not utilized in this study. The original full and brief versions include a 6th question in addition to the five items on prosocial reasoning for each test scenario. The items were removed as many expert panel members regarded it as not necessary and confusing to potential respondents. However, we believe it is likely that social desirability can greatly influence responses to a test of prosocial reasoning. Future studies should try to include these items and estimate how far respondents are trying to tell people they are prosocial.

In summary, the results indicate that the Chinese PROM had good-content validity and reliability. The quality of translation, cultural relevance, and reading level have been satisfactorily revised for use with adolescents. There is, however, partial support to the convergent validity of the Chinese PROM. The PROM scores had high positive correlations with empathy and negative correlations with personal distress and fantasy. These results were consistent with theoretical deductions, although it is also a concern that empathy had a close-to-unity correlation with PROM score in the small sample study of stage 1. The relationship between PROM scores and prosocial behavior tends to be weak. It appears that there are many personal, family, or social factors that are linked to prosocial behavior, and prosocial reasoning may only contribute to a small proportion of variation in prosocial behavior among adolescents.

## Figures and Tables

**Table 1:** Reliability study of the Chinese PROM.

PROM scale and subscales			
	Test-retest (ICC) (*n* = 25)	Internal consistency	
		This study (*n* = 50)	Carlo [10] (*n* = 27)
Hedonistic	.83	.91	.72
Needs oriented	.88	.93	.56
Approval oriented	.75	.74	.78
Stereotypic	.81	.89	.67
Internalized	.88	.93	.70
PROM weighted total	.88	.89	#

^#^Not reported.

**Table 2:** Correlation between the Chinese PROM and the C-IRI (measure of empathy and related constructs) (*N* = 50).

PROM scales and subscales	Chinese C-IRI subscales		
	Fantasy scale	Empathy scale	Personal Distress scale
Hedonistic	.55 **	−.78 **	.61 **
Needs oriented	−.71 **	.72 **	−.72 **
Approval oriented	.68 **	−.76 **	.69 **
Stereotypic	−.67 **	.77 **	−.65 **
Internalized	−.78 **	.92 **	−.82 **
PROM weighted overall	−. 77 **	.92 **	−.80 **

*P < .05, **P < .01.

**Table 3:** Correlation between prosocial moral reasoning and prosocial/antisocial behavior in adolescents (*N* = 566).

PROM subscales and Weighted overall	Antisocial behavior	Prosocial behavior	Adolescent behavior
Hedonistic	.14**	−.10*	−.17**
Need oriented	.05	.06	.04
Approval oriented	−.09	−.04	.00
Stereotypic	−.06	.04	.07
Internalized	−.06	.07	.10*
Overall Weighted	−.05	.10*	.12*

*P < .05, **P < .01.

**Table 4:** Comparison of prosocial reasoning between males and females, with age as covariate.

	Male^a^		Female^a^		
Variables	(*n* = 182)		(*n* = 379)		***F***
	*M*	*SE*	*M*	*SE*	

Hedonistic	.176	.002	.168	.001	13.06***
Needs oriented	.206	.002	.212	.001	7.54**
Approval oriented	.183	.002	.184	.001	.49
Stereotypic	.219	.002	.220	.001	.00
Internalized	.216	.001	.216	.001	.08
PROM overall weighted	1.86	.004	1.86	.003	2.33

*P < .05, **P < .01, ***P < .001.

^a^Estimated marginal means and standard errors were presented, adjusted for age effects.
